# Pembrolizumab in FIGO IVB Verrucous Carcinoma of the Vulva: A Case Report

**DOI:** 10.3389/fonc.2021.598594

**Published:** 2021-05-28

**Authors:** Yuhan Wang, Rongchun Lin, Bingzhong Zhang, Hui Zhou, Zhongqiu Lin, Tingting Yao

**Affiliations:** ^1^ Department of Gynecological Oncology, Sun Yat-sen Memorial Hospital, Sun Yat-sen University, Guangzhou, China; ^2^ Key Laboratory of Malignant Tumor Gene Regulation and Target Therapy of Guangdong Higher Education Institutes, Sun Yat-sen University, Guangzhou, China

**Keywords:** vulva cancer, verrucous carcinoma, immunotherapy, chemotherapy, pembrolizumab

## Abstract

**Background:**

Vulvar cancer is the fourth most common gynecologic cancer, and prognosis is poor in advanced vulvar cancer patients. Treatment for advanced vulvar cancer has not been satisfactory. In this report, we firstly report a FIGO IVB vulva verrucous carcinoma patient who obtained good prognosis after systemic treatment.

**Case Presentation:**

A patient was admitted to hospital due to her vulvar lesion persistent for past 14 years. The vulvar mass has widely invaded urethra, part of anus, the lower third of the vagina, bilateral superior and inferior branches of pubis, and bilateral internal and external muscles of obturator. Multiple metastatic lymph nodes were also found in the pelvic cavity. The histopathological studies confirmed vulvar verrucous carcinoma with a PD-L1 overexpression. After six courses of neoadjuvant chemotherapy and pembrolizumab, the patient underwent radical vulvectomy and achieved optimal cytoreduction. Postoperative pathology found no residual tumor. The patient then received one course of postoperative chemotherapy and pembrolizumab, underwent radiation therapy, and was disease free after 6 months follow-up.

**Conclusion:**

Our individualized treatment strategy is successful. Pembrolizumab is safe and effective in the treatment of advanced vulvar verrucous carcinoma with PD-L1 overexpression.

## Background

Vulvar cancer is the fourth most common gynecologic cancer, accounting for 3.6% of all malignancies of the female genital system (1). Surgery forms the cornerstone of management of vulvar cancer (2). However, the treatment focus often shifts to palliative chemoradiotherapy for stage IVB patients, because an extensive excision of the disease is usually not feasible (3). The prognosis is poor for stage IV vulvar cancers, with 1-year and 5-year survival rates of 37.5% and 12.5%, respectively (4). The treatment of advanced vulvar cancer remains a major challenge.

In recent years, immunotherapy has been widely used, especially in advanced and metastatic cancers. PD-1/PD-L1 pathway plays an important role in immune regulation. Programmed cell death 1 ligand 1 (PD-L1) is an immunoinhibitory molecule mainly expressed on the surface of tumor cells and antigen-presenting cells in various malignant tumors. Programmed cell death 1 (PD1), a PD-L1 receptor, is expressed on the surface of T cells, B cells, monocytes, and macrophages. The binding of PD-L1 and PD-1 causes the exhaustion of effector T cells and immune escape of tumor cells, leading to poor prognosis (5). Pembrolizumab is a humanized monoclonal antibody against PD-1, stopping the tumor from evading the immune response. It has been successfully used in patients of different cancers, including a patient with recurrent vulvar cancer (6). In this report, we present the first case of a 41-year-old patient with advanced vulvar verrucous carcinoma who was treated with pembrolizumab.

## Case Presentation

**Figure 1:**
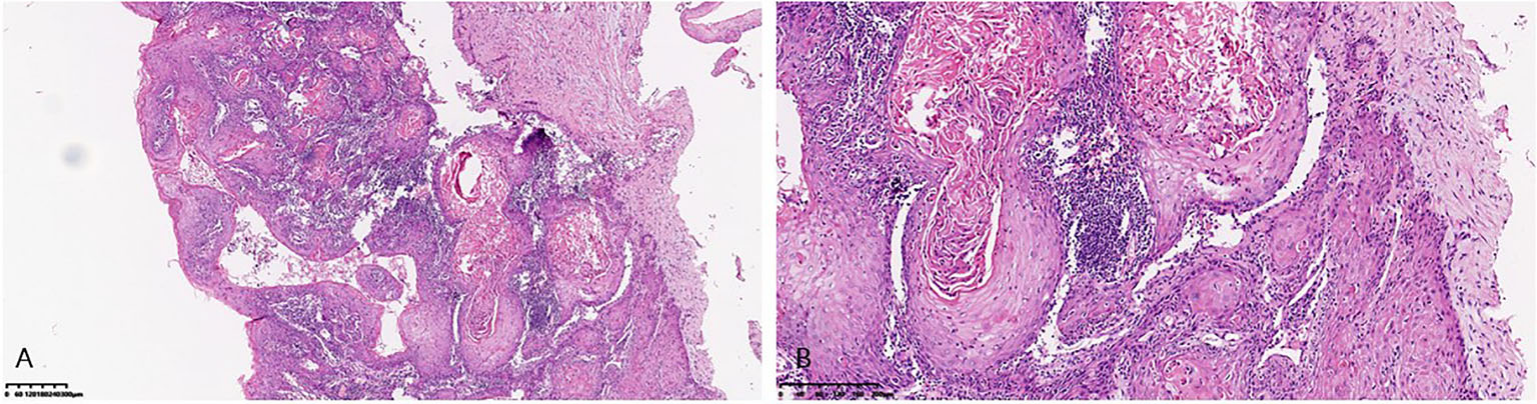
Microscopic pattern of verrucous carcinoma of the vulva. **(A)** Low-power view of verrucous carcinoma of the vulva showing nests and islands of well- differentiated squamous epithelial cells embedded in a dense stroma. **(B)** High-power view showing extremely well- differentiated squamous epithelial cells.

A 41-year-old patient presented to us due to a vulvar lesion present for the last 14 years. She visited several gynecologists and only received symptomatic treatment without pathology confirmation. In December 2017, the patient was conscious of swelling of the vulva and mons pubis, she underwent a biopsy and was diagnosed as condyloma acuminatum of vulva. From January 2018 to November 2018, the patient experienced recurrent inguinal lymphadenectasis with fever. The computed tomography (CT) imaging showed that the density of lesion in the left perineum was not uniform, and the lesion was obviously strengthened unevenly after enhancement. The necrotic areas of the lesion were not strengthened, enlarged lymph nodes were found in the groin areas on both sides. The patient was considered to have perineum abscess with suspected sinus formation. She was discharged after receiving anti-infection treatment, incision and drainage of right inguinal region abscess, and vacuum sealing drainage. However, in January 2019, the patient again developed right inguinal lymphadenectasis. She received bilateral inguinal enlarged lymph node biopsy, left labia mass biopsy and right inguinal debridement and drainage. The histopathological studies confirmed the presence of vulva verrucous carcinoma, and metastatic highly differentiated squamous cell carcinoma was found in the removed lymph nodes (1/1). According to the 2009 International Federation of Gynecology and Obstetrics (FIGO) staging criteria for vulvar carcinoma, the tumor was classified as stage IVB. At this time, a re-examination of the pathology in 2017 revealed that the pathology at that time also suggested verrucous carcinoma (**Figure 1**). The patient underwent positron emission tomography/computed tomography (PET/CT) in January 2019. It showed that the vulvar mass, considering to be vulvar cancer, widely invaded urethra, part of anus and the lower third of the vagina. Multiple metastatic lymph nodes were found in the pelvic cavity, adjacent to bilateral iliac vessels and bilateral inguinal region. Bilateral superior and inferior branches of pubis were metastasized, and bilateral internal and external muscles of obturator were invaded.

**Figure 2:**
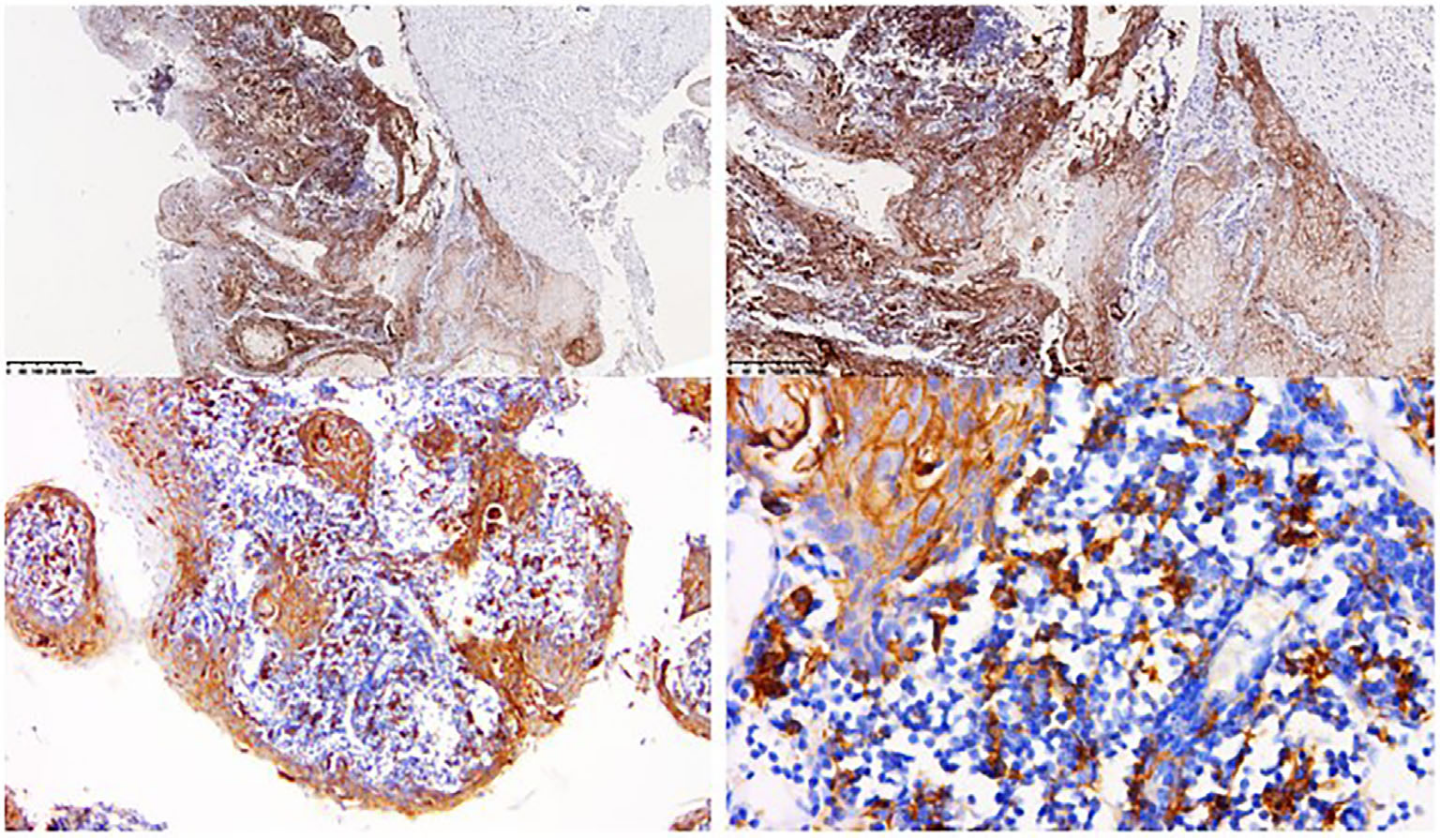
Immunohistochemical testing of tissue from the first biopsy. Immunohistochemical testing of tissue from the first biopsy reported strong membranous staining of PD-L1 in more than 90% of tumor cells and 25% of tumor associated immune cells.

She came to our gynecologic oncology clinic for future treatment in March 2019. On clinical examination, an exophytic tumor of 10 cm in maximum was involving urethra and anterior vaginal wall. The magnetic resonance imaging (MRI) scan from the time of admission was similar to previous imaging. Tumor biomarker squamous cell carcinoma antigen (SCCA) was >100 ng/ml, HPV test was negative. Immunohistochemical testing of tissue from the first biopsy reported a PD-L1 overexpression (**Figure 2**). She received neoadjuvant chemotherapy and immunotherapy, consisting of paclitaxel-albumin 300 mg, cisplatin 100 mg, and pembrolizumab 100 mg. Considering the relatively high neurotoxicity of cisplatin and the patient’s history of congenital spina bifida, the therapy regimen was changed to docetaxel 90 mg, carboplatin 500 mg, and pembrolizumab 100 mg from her third treatment. The patients then received four more courses of neoadjuvant chemotherapy and immunotherapy.

**Figure 3:**
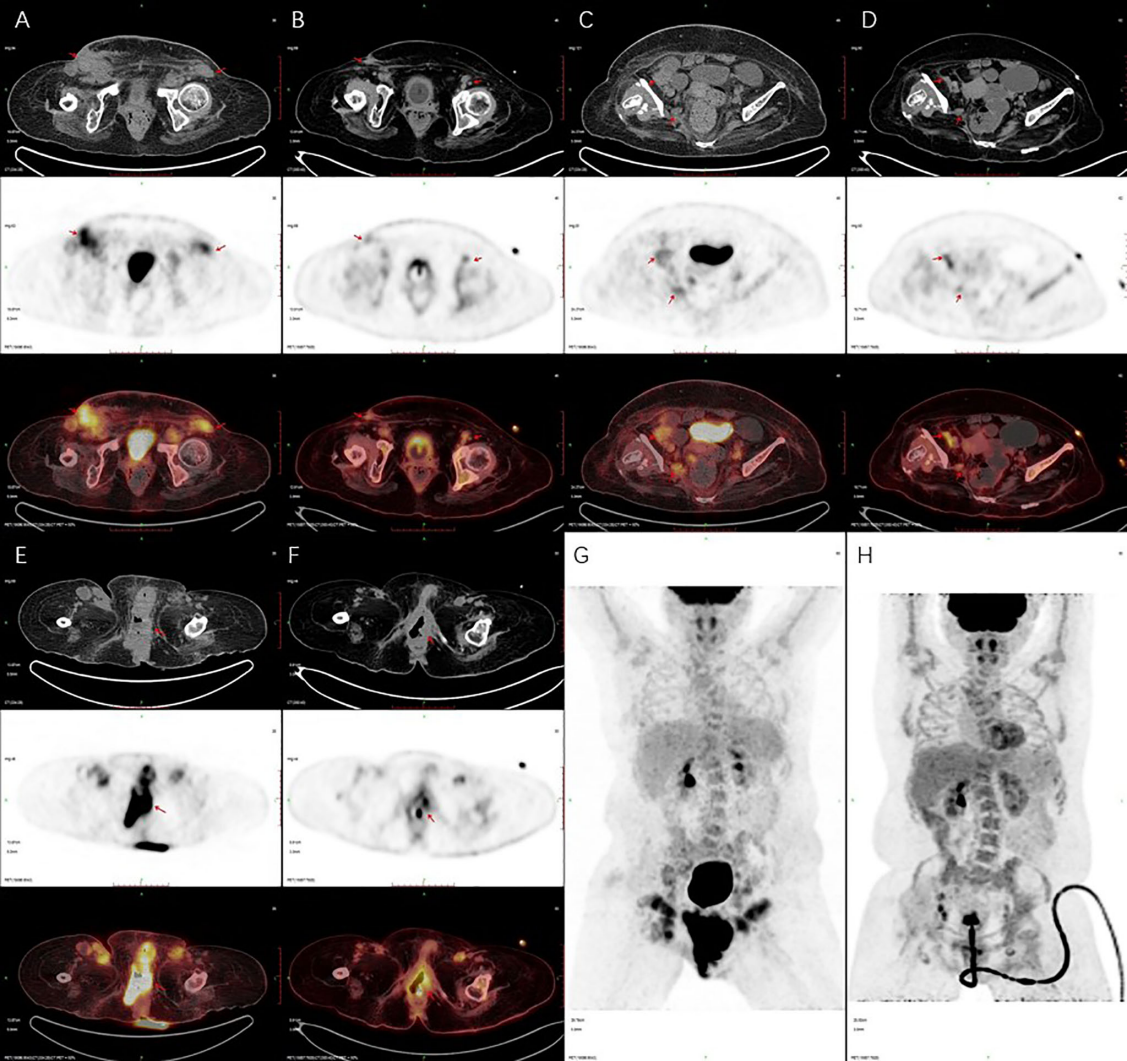
Positron emission tomography/computed tomography (PET/CT) imaging. PET/CT scan showed that metastatic lymph nodes were smaller and less than before **(A–D)**, and the vulva lesions were smaller than before **(E–H)**. **(A, C, E, G)** are PET/CT imaging taken in January 2019. **(B, D, F, H)** are PET/CT imaging taken in October 2019.

**Figure 4:**
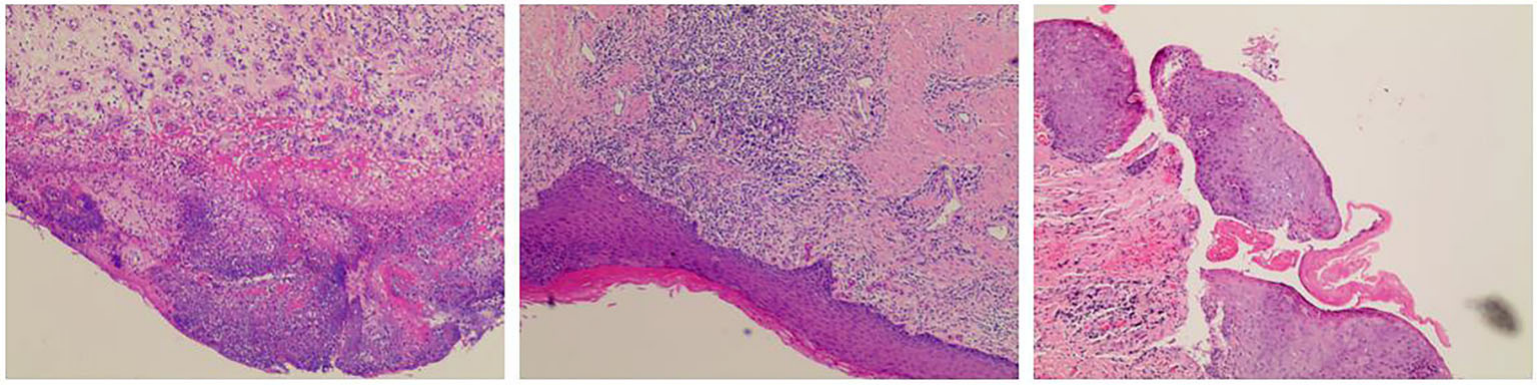
Postoperative pathological. Pathological examination revealed a vulva low-grade squamous intraepithelial lesion (LSIL) with negative margin, no residual cancer was found.

In October 2019, after a total of six courses of neoadjuvant chemotherapy and immunotherapy, the PET/CT scan showed that the lesions were smaller than before, metastatic lymph nodes were smaller and less than before (**Figure 3**). Then the patient underwent radical vulvectomy and vulvoplasty. The surgical scope was cut from the outer 2 cm of the tumor, the upper boundary to the outer edge of the clitoris 2 cm, the lower boundary to the front of the anus, and the inner boundary to the level of the hymen, the tumor and subcutaneous tissue were completely removed, and the resection depth was up to the level of the deep fascia. Pathological examination of the specimen confirmed a vulva low-grade squamous intraepithelial lesion with negative margin, no residual cancer was found (**Figure 4**).

The patient received one course of docetaxel 90 mg, carboplatin 500 mg, and pembrolizumab 100 mg after surgery. After postoperative immunotherapy, the patient developed severe arthralgias in her right wrist and elbow. She visited the department of rheumatology and was considered to have immunotherapy-induced immune arthritis, which was significantly relieved by the application of methylprednisolone and methotrexate. Between December 2019 and February 2020, the patient received pelvic radiotherapy, comprising PGTVn 60Gy/27F and PTV 48.6Gy/27F. This patient was disease free after 6 months follow-up, and her anatomical and functional prognosis are good.

## Discussion

Verrucous carcinoma is a rare type of vulvar cancer with characteristics of slow-growing, less metastasis but frequently expanding (7, 8). Because of these characteristics, vulva verrucous carcinoma cases reported so far are mostly in early stages with giant size. A literature review of 2019 that included 19 verrucous carcinoma of vulva patients found that more than 90% of patients are in the early stages (FIGO stage I and II) (9). This case is the first stage IVB vulva verrucous carcinoma case that has ever reported.

The exact pathogenesis of verrucous carcinoma of vulva remains unclear. Though human papillomavirus (HPV) infection has been considered as a risk factor for the pathogenesis of this tumor (10, 11), the role for HPV is doubtful (9). Usually, this rare type of pathology occurs in elderly postmenopausal women, premenopausal women with this tumor often have HPV infection (8, 12). This patient is non-menopausal, but her tumor is not HPV related. Accurate diagnosis of verrucous carcinoma is sometimes difficult, because this rare pathological type is easily misdiagnosed as condyloma, both macroscopically and microscopically (12). The external part of verrucous carcinoma is similar to condyloma acuminatum in histopathology, the difference between the two lesions lies in the pathological manifestations of the base part of the tumor. However, small and superficial verrucous carcinoma are often not easy to distinguish with condyloma acuminatum because of the specimen obtained by biopsy without or with only a small base part. Therefore, gynecologic oncologist and pathologist should recognize the presence of this rare pathological type, pay attention to remove the specimen from the base part of the tumor and distinguish it from condyloma and other tumors.

Treatment options for patients with vulvar verrucous cancer remain controversial, with no consensus on a standard regimen. The treatment of early stage vulvar verrucous cancer tends to be more conservative recently, the wide local excision is considered to be the best treatment (8). It is crucial to perform an extensive excision of the disease, because of the risk of local relapse after inadequate surgery (13). It is recommended that a free surgical margin of at least 1 cm should be achieved at primary surgery to avoid recurrences (14). While the treatment of advanced vulvar verrucous cancer is rarely reported.

Radiation therapy is often used in the management of patients with advanced vulvar cancer (14). However, radiotherapy for verrucous carcinoma is doubtful, because high reoccurrence rates with radiotherapy have been observed (15). Besides, it has been found that radiotherapy may induce anaplastic transformation (16), but this finding has not been totally accepted yet. Due to relatively poor general condition of this patient, pelvic lymph node dissection was not performed, so the patient received postoperative pelvic radiotherapy. Pathological transformation after radiotherapy was not seen in this case and the prognosis is acceptable. We think postoperative radiotherapy may be beneficial for the treatment of advanced vulva verrucous carcinomas, especially for those without pelvic lymph node dissection.

With the deepening understanding of the molecular basis of tumor cell immune recognition and immune regulation, immunotherapy has attracted great interest in recent years. Immunotherapy includes immune checkpoint blockade, cancer vaccines, and adoptive cell therapy (17). PD-L1 and PD-1 constitute a regulatory immune checkpoint, which plays an important role in self-tolerance. The binding of PD-L1 and PD-1can inhibit the activation of T cells, leading to immune escape of tumor cells, progression of tumors, and poor prognosis (5). PD-1/PD-L1 inhibitors therapies has been used in the treatment of many cancers, especially in advanced and metastatic cancers. In 2018, a report presented the first case of a patient with recurrent vulvar cancer who was treated with pembrolizumab, proving the safety and efficacy of pembrolizumab in the treatment of recurrent vulvar carcinoma (6). The 2020 National Comprehensive Cancer Network (NCCN) Guidelines recommend pembrolizumab as a second-line therapy for PD-L1 positive or MSI-H/dMMR tumors. In this case, when the patient first came to our gynecologic oncology clinic, her lesion was very extensive and widely involved peripheral tissues, local excision was not possible. So, we advised the patient to receive neoadjuvant chemotherapy and immunotherapy first. We determined the best therapy regimen for the patient, which comprising docetaxel 90 mg, carboplatin 500 mg, and pembrolizumab 100 mg. After a total of six courses of neoadjuvant chemotherapy and immunotherapy, the PET/CT scan showed that the lesions were smaller than before, then the patient underwent radical vulvectomy and achieved optimal cytoreduction. Postoperative pathology found no residual tumor, indicating that neoadjuvant chemotherapy and immunotherapy had a good effect. The patient received one course of chemotherapy and immunotherapy, and underwent pelvic radiotherapy after the surgery. She was disease free after 5 months follow-up with good anatomical prognosis and functional prognosis, which means our therapeutic schedule is successful. Immunohistochemical testing of tissue from the first biopsy reported strong membranous staining of PD-L1 in more than 90% of tumor cells, supporting that pembrolizumab is effective for PD-L1 positive tumors.

Several toxicities have been encountered with pembrolizumab, such as fatigue, nausea, myalgia, arthralgia, and colitis (6, 18, 19). In this case, though the patient developed severe arthralgias in her right wrist and elbow, and it can be relieved by the application of methylprednisolone and methotrexate.

## Conclusion

Vulvar cancer is a rare gynecologic cancer. The treatment of stage IVB vulvar cancer often focuses on palliative chemoradiotherapy, because an extensive excision is usually not feasible, and the prognosis is poor in these patients.

The patient in this study was diagnosed with stage IVB vulva verrucous carcinoma, extensive excision was not possible when she first came to our center. However, the patient achieved optimal cytoreduction after neoadjuvant chemotherapy and immunotherapy. Postoperative pathology found no residual tumor, and she was disease free after 6 months follow-up with good anatomical and functional prognosis. The outcome of this patient demonstrates that the treatment regimen we developed for her is effective, and revealed the efficacy and safety of pembrolizumab in the treatment of advanced vulvar verrucous carcinoma with PD-L1 overexpression.

## Ethics Statement

Written informed consent was obtained from the individual(s) for the publication of any potentially identifiable images or data included in this article.

## Author Contributions

YW, RL, BZ, HZ, ZL, and TY all participated in the care of the patient and wrote the first draft of the manuscript. TY helped prepare the pathology text, pathology figures, and the pathology figure legends. YW, ZL, and TY took part in the investigations for the patient. All authors had full access to the data and vouch for their integrity and accuracy. All authors contributed to the article and approved the submitted version.

## Funding

This work was supported by the Natural Science Foundation of Guangdong Province (2016A030310178).

## Conflict of Interest

The authors declare that the research was conducted in the absence of any commercial or financial relationships that could be construed as a potential conflict of interest.

The reviewer LZ declared a shared affiliation, with no collaboration, with the authors to the handling editor at the time of the review.

## Abbreviations

PD-1, programmed cell death 1; PD-L1, programmed cell death ligand 1; CT, computed tomography; FIGO, International Federation of Gynecology and Obstetrics; PET/CT, positron emission tomography/computed tomography; MRI, magnetic resonance imaging; SCCA, squamous cell carcinoma antigen; HPV, human papillomavirus.
